# *Salmonella* Derby: A Comparative Genomic Analysis of Strains From Germany

**DOI:** 10.3389/fmicb.2021.591929

**Published:** 2021-05-24

**Authors:** Belén González-Santamarina, Silvia García-Soto, Helmut Hotzel, Diana Meemken, Reinhard Fries, Herbert Tomaso

**Affiliations:** ^1^Institute of Bacterial Infections and Zoonoses, Friedrich-Loeffler-Institut, Jena, Germany; ^2^Institute of Institute of Molecular Pathogenesis, Friedrich-Loeffler-Institut, Jena, Germany; ^3^Institute of Food Safety and Food Hygiene, Section Meat Hygiene, Freie Universität Berlin, Berlin, Germany

**Keywords:** Germany, pig, antimicrobial resistance, sequence type, whole-genome sequencing, *Salmonella* Derby

## Abstract

*Salmonella enterica* subspecies *enterica* serovar Derby (*S*. Derby) is one of the most frequent causes for salmonellosis in humans and animals. Understanding the genetic diversity of *S*. Derby, as well as the nature and origin of its resistance to antimicrobial treatment are thus the key to epidemiological control and surveillance. Here, we report an analysis of 15 *S.* Derby strains isolated from pig and cattle in slaughterhouses across Germany (2000–2015), which belonged to multilocus sequence types (ST) ST39, ST40 and ST682. Strains were compared to publicly available *S*. Derby sequence data of these three STs from Germany, comprising 65 isolates collected between 2004 and 2018 from different sources (i.e., pigs, humans, cattle, wild boar, and poultry). A total of 80 sequences (ST39 = 34, ST40 = 21, and ST682 = 25) were analyzed to assess genetic diversity, to identify virulence-associated and antimicrobial resistance genes (ARGs), and to characterize plasmid content. Strains belonging to all three STs were identified in each source examined. Strains with the same ST were closely related regardless of origin. Altogether, 72.5% of the isolates carried at least one resistance gene, furthermore ST40 carried most of the ARGs and the plasmid replicons. The IncI1 replicon was detected in eleven isolates, four of them carried IncI1 plasmid ST26 with clonal complex 2. The comparison of these four isolates with an IncI1 ST26 plasmid reported in 2010 from a German pig (JX566770), showed only variations in a region carrying different ARGs and mobile genetic elements. The strains of our collection had similar genetic diversity as the strains taken from the public database. Moreover, we found that strains harboring multidrug resistant IncI plasmid were found in different animal species, indicating that S. Derby may be implicated in the spread of antimicrobial resistance among animal species. Results may contribute to the knowledge about the diversity in *S.* Derby in Germany, which may be useful for the future surveillance and antimicrobial resistance of this serovar.

## Introduction

*Salmonella* spp. (*S.*) is the second most frequently reported zoonotic bacterial agent after *Campylobacter* (EFSA, 2019) in the European Union (EU). The most common source of foodborne outbreaks were eggs, poultry meat, and pork (EFSA, 2019).

*Salmonella enterica* subspecies *enterica* serovar Derby has its highest prevalence in pigs and cattle. In pigs, despite frequent isolation of *S.* Derby, it does not cause significant enteric disease (Naberhaus et al., 2019). However, *S*. Derby can cause acute enteric salmonellosis in cattle and abortions in pregnant cows (Barrow and Methner, 2013). Between 2013 and 2018, this serovar ranked fourth to fifth cause of *Salmonella* outbreaks in humans in Germany. Moreover, it is the most common serovar isolated from infants and toddlers in China, the largest pork producer and consumer in the world (Stanaway et al., 2019). In the United States, this serovar ranks as the ten most frequently isolated serovars in humans (Hauser et al., 2011).

In the EU, multidrug resistance (MDR) *S.* Derby isolates were found in 11.8% of pig carcasses samples and in 15.3% of pig’s isolates (EFSA, 2019). In Germany, the prevalence in 2017 was 2.5% in pig carcass samples and 7.1% in pigs (BVL, 2018). The most common resistance pattern included resistance against sulfamethoxazole, trimethoprim, and tetracycline. In calf carcasses under one year of age, a single S. Derby isolate (1/7, 14.3%) exhibited MDR (chloramphenicol, sulfamethoxazole, and trimethoprim) (EFSA, 2019). High colistin resistance (MICs of ≥ 16mg/L) was found in two *S.* Derby isolates in Germany, and the only resistance to third-generation cephalosporin was detected in a single *S.* Derby isolate recovered from pig carcasses in Germany (EFSA, 2019). Furthermore, in this country between 2006 to 2008, 25% of the S. Derby isolates taken from pig and human carried antimicrobial resistance genes (ARG) such as *tetB, sul1, aadA2*, and *int1* (Hauser et al., 2011).

Whole-genome sequencing (WGS) of *S.* Derby has been reported in France (Sévellec et al., 2018), Germany (Hauser et al., 2011; Simon et al., 2018), the United States (Pornsukarom et al., 2018) and China (Zheng et al., 2017). These studies used multilocus sequence typing (MLST) to differentiate between strains and to elucidate the host specificity of sequence types (ST) in *S.* Derby (Zheng et al., 2017; Simon et al., 2018; Sévellec et al., 2018). While the study (Sévellec et al., 2018) reports in detail on diversity and antimicrobial resistance of *S.* Derby in France, a similarly exhaustive WGS study is still lacking in Germany.

Between 2014 and 2018, poultry-specific ST71 was the most frequently recorded ST in the Enterobase *Salmonella enterica* MLST database^1^. Other important STs predominantly associated with pork were ST40 and ST39. ST682 was observed in a national outbreak in Germany in 2013 (Simon et al., 2018). The Senterica database also contains many more *S*. Derby STs, such as ST15, ST678, ST683, ST695, ST774, ST813, ST1326, ST3871, ST72 and ST1585 (Zheng et al., 2017).

In this study, we compare 15 *S*. Derby isolates with a collection of 65 public sequence data from different sources (i.e., pig, human, cattle, wild boar, and poultry) in Germany using WGS for epidemiological analysis and the characterization of resistance and virulence. Besides, we examined the diversity of plasmids and their role in the spread of antibiotic resistance. The comparison of our strains with the strains from public databases shows similarities and thus provides more information regarding genetic diversity. Therefore, our results provide further data on antimicrobial resistance of *S*. Derby in Germany as well as its distribution in different animal species and humans.

## Materials and Methods

### Bacterial Isolates

This study analyzed a collection of 15 *S.* Derby available isolates collected from five different pig and cattle slaughterhouses from 2000 to 2015 in Germany (Table 1). The samples were taken from cattle (minced meat), pigs (carcasses, mesenteric lymph nodes, and rectum), and the environment (slaughterhouse’s pen, corridor, and truck driver’s boots) (Table 1). The isolates belonged to the strain collection of the retired Univ.-Prof. Dr. Reinhard Fries. They were isolated, identified and serotyped following the DIN EN ISO 6579-1:2017-07 between 2000 and 2015 as part of usual laboratory diagnosis and a previously published study (Gonzalez Santamarina, 2019). The strains were stored in glycerol or cryotubes at −20°C. In July 2019, they were transferred from the Institute of Food Safety and Food Hygiene, Working Group Meat Hygiene (Freie Universität Berlin) to the Institute of Bacterial Infections and Zoonoses (IBIZ) (Jena) without interruption of the cold chain.

**TABLE 1:** *Salmonella* Derby isolates of our collection.

Slaughterhouse	Year	Origin	N
1	2000	19CS0432	Cattle (Minced meat)
2	2001	19CS0435	Cattle (Minced meat)
3	2005	19CS0409	Pig
		19CS0438	
	2006	19CS0453	Pig
	2008	19CS0454	Pig
	2010	19CS0413	Pig Carcass
		19CS0427	
	2011	19CS0429	Pig Carcass
		19CS0430	
4	2014	19CS0456	Pig Mesenteric lymph nodes
5	2015	19CS0462	Pig Rectum
		19CS0459	Pig Slaughterhouse’s pen
		19CS0460	Pig Slaughterhouse’s corridor
		19CS0458	Pig Driver truck boots

Cultures were inoculated into 3 ml of brain-heart infusion broth, and incubated between 4 and 18 h at 37°C. They were cultivated on RAMBACH^®^ Agar (Merck KGaA, Darmstadt, Germany) for 24 ± 3h at 37°C. The typical *Salmonella* spp. colonies with a red-pink color and a bright edge were then taken for further bioinformatics analysis.

### Whole-Genome Sequencing

To perform next-generation sequencing, the fifteen strains were grown overnight at 37°C in 3 ml of Luria-Bertani broth (Mast Diagnostica GmbH, Reinfeld, Germany). The genomic DNA was extracted and purified using the QIAGEN^®^ Genomic-tip 20/G kit (QIAGEN, Germany) and the Genomic DNA Buffer Set (QIAGEN, Germany). The concentration of the DNA was determined using the Qubit dsDNA BR assay kit (Invitrogen, United States). DNA sequencing libraries were constructed using the Nextera XT Preparation Kit (Illumina Inc., San Diego, CA, United States) following the manufacturer’s instructions. Paired-end sequencing was performed on the Illumina MiSeq platform (Illumina Inc., San Diego, CA, United States) using a 300-cycle MiSeq reagent kit.

### Bioinformatics Analyses

We performed bioinformatics analysis of the strains using an in-house Linux-based bioinformatics pipeline WGSBAC v.2.0 (available at https://gitlab.com/FLI_Bioinfo/WGSBAC/-/tree/version2). The WGSBAC pipeline took Illumina raw reads as input and assessed their quality using FastQC v0.11.7 (available at http://www.bioinformatics.babraham.ac.uk/projects/fastqc/) and coverage was calculated using an adapted script^2^. Reads were *de novo* assembled using Shovill (v. 1.0.4) (Seemann, 2018) and evaluated with QUAST v. 5.0.2 (Gurevich et al., 2013) in standard settings. Annotation was performed with Prokka (v.1.14.5) (Seemann, 2014). The pipeline used Kraken 2 (v. 1.1) to identify contamination, and the database Kraken2DB to classify both reads and contigs (Wood et al., 2019). Genes coding for resistance and point chromosomal mutations were detected using the software AMRFinderPlus (v. 3.6.10) (Feldgarden et al., 2019). Additionally, Abricate v.0.8.10 (available at https://github.com/tseemann/abricate) with the databases ResFinder (v. 3.2) (Zankari et al., 2012), CARD (v. 3.0.8) (Jia et al., 2017) and NCBI are used for resistance gene detection. Abricate was also used in conjunction with the Virulence Factor Database (VFDB) (Chen et al., 2005; Liu et al., 2019) and PlasmidFinder (v.2.0.1) (Carattoli and Hasman, 2020) for the prediction of virulence-associated genes and plasmids replicons, respectively. The pMLST 2.0 (v. 1.3) (Carattoli and Hasman, 2020) server which contains the Plasmid PubMLST database^3^ (Carattoli and Hasman, 2020) was used for plasmid typing.

For genotyping, WGSBAC used classical multilocus sequence typing (MLST) (v. 2.16.1) on assembled genomes with automatic scheme detection and an assembly-based genotyping using whole-genome assemblies with ParSNP (v.1.2) from the HARVEST-Suite. Mapping-based SNP-typing was performed by Snippy (v. 4.3.6) which output (SNPs distance matrix) is used to build SNP-based phylogenetic trees by FastTree (v. 2.1.10) (Price et al., 2009).

For *in silico* serotyping detection the WGSBAC pipeline used SISTR (v1. 0.2) (Yoshida et al., 2016). For the detection of *Salmonella* pathogenicity islands (SPIs), we used a combination of an external software SPIFinder (1.0) (Roer et al., 2016) and a customized database built by downloading public SPIs sequences from NCBI database. Visualization of phylogenetic trees was carried out by the online tool iTOL (v. 5.1 0) (Letunic and Bork, 2019). The program Geneious prime (version 11.1.5) (Biomatters, Ltd., Auckland, 1010, New Zealand) was applied for visualization and alignment of sequences.

### Database Comparison

For this study, we constructed a database of isolates that: (i) were collected in Germany; (ii) had been identified as *S.* Derby; (iii) belonged to the same sequence types found in our samples (ST39, ST40, and ST682). In total, sequence data of 65 bacterial isolates were taken from two different studies. 39 sequences belonging to the Istituto Zooprofilattico Sperimentale delle Venezie, were downloaded from NCBI with accession number PRJEB23440^4^. These strains were part of a study that characterized *S.* Derby isolates from different sources, with focus on samples collected from pork in Germany and Italy. Another 26 sequences (PRJEB30317), which had been provided by the Robert-Koch-Institute (RKI), were downloaded from the European Nucleotide Archive (ENA)^5^. All the sequence data carried information on source and year of collection. However, information about the exact geographical location of the collection was not available (Supplementary Table 1). Therefore, this study provides more information about the diversity of *S*. Derby in Germany; it does not claim to be representative for this serotype or its distribution within the country.

## Results

### Serovar Prediction and MLST Typing

Using *in silico* serotype detection with SISTR (v1. 0.2), all samples of both collections were confirmed as serovar *Salmonella* Derby with antigen formula 1,4,5 [12]:f,g:- according to the White-Kauffmann-Le Minor scheme (Grimont and Weill, 2007). Three different sequence types (STs) were found: ST39 (*aroC*(19), *dnaN*(20), *hemD*(3), *hisD*(20), *purE* (5), *sucA*(2) and *thrA*(22)), ST40 (*aroC* (19), *dnaN* (20), *hemD* (3), *hisD* (20), *purE*(5), *sucA* (22) and *thrA* (22)) and ST682 (*aroC* (45), *dnaN* (60), *hemD* (12), *hisD* (237), *purE* (193), *sucA* (2)and *thrA* (96)).

In the FLI collection, most common ST was ST39 with 60% (9/15) of the total strains, followed by ST40 26.6% (4/15) and ST682 13.3% (2/15).

In the public collection, ST39 was present in 38.5% (25/65) of the sequences. ST682 was found in 35.4% (23/65) of the isolates followed by ST40, which was found in 26.15% (17/65) of the sequences.

### Antibiotic Resistance Genes

In total, 23 genes conferring resistance to antibiotics were found (Supplementary Table 2). 72.5% (58/80) of the isolates carried at least one antimicrobial resistance gene (ARG). Genes conferring resistance against fosfomycin (*fosA7, fosA7.3)* were most frequently found; 68.7% (55/80) of the strains carried one of these genes. It was followed by other ARGs against sulfonamide (*sul1, sul2, sul3*) found in 17.5% of the strains (14/80), aminoglycosides (*aadA1, aadA2, aph(6)-Id, aph(3”)-Ib*) in 13.7% (11/80) and tetracycline (*tetB, tetM, tetG, tetA*) in 11.2% (9/80). Genes associated with resistance to β-lactam-antibiotics (*bla*_TEM–1_) were found in 8.7% (7/80) of the isolates followed by trimethoprim (*dfrA14, dfrA1, dfrB1*) 7.5% (6/80) and phenicol (*cmlA1, floR, catB2*) 5% (4/80). Quinolone resistance (*qnrS1, qnrB19*) was found only in 3.7% (3/80) of the strains and one isolate carried a mutation in the gyrase-encoding gene (*gyrA_S83F*).

In the FLI collection, all ST39 isolates (Table 2) carried *fosA7*. Only one sample, 19CS0430 (pig), carried other ARGs (*bla*_TEM–1_, *aadA1, aadA2, tetA, sul3*, and *cmlA1).* No ARGs to quinolones were found in this ST. In ST40, *fosA7.3* was present in all the isolates (Table 3) and two of them carried *qnrS1.* ST682 isolates did not carry any resistance genes (Table 4).

**TABLE 2:** ST39 genome information about the antimicrobial resistance genes, plasmid replicons and virulence genes, *n* = 34.

Metadata				Antibiotic resistance genes		Plasmid replicon		Virulence Genes	
Year	ID strain	Collection	Sample	N	Gene names	N	Replicon name	N	genes
2001	19CS0435	FLI	Cattle	1	*fosA7*	0		125	*cheADWY, entCES, fepABE, flgGH, fliACGMNP, gmhA.lpcA, gtrAB, iroBCN entA, ratB, shdA, sspH2, shdA, steB sopD*
2005	19CS0409	FLI	Pig	1	*fosA7*	1	ColRNA_1	125	*cheADWY, entCES, fepABE, flgGH, fliACGMNP, gmhA.lpcA, gtrAB, iroBCN entA, ratB, shdA, sspH2, shdA, steB sopD*
2005	19CS0438	FLI	Pig	1	*fosA7*	1	ColRNAI_1	125	*cheADWY, entCES, fepABE, flgGH, fliACGMNP, gmhA.lpcA, gtrAB, iroBCN entA, ratB, shdA, sspH2, shdA, steB sopD*
2006	19CS0453	FLI	Pig	1	*fosA7*	1	ColRNAI_1	125	*cheADWY, entCES, fepABE, flgGH, fliACGMNP, gmhA.lpcA, gtrAB, iroBCN entA, ratB, shdA, sspH2, shdA, steB sopD*
2008	19CS0454	FLI	Pig	1	*fosA7*	1	ColRNAI_1	125	*cheADWY, entCES, fepABE, flgGH, fliACGMNP, gmhA.lpcA, gtrAB, iroBCN entA, ratB, shdA, sspH2, shdA, steB sopD*
2011	19CS0429	FLI	Pig	1	*fosA7*	1	ColRNAI_1	125	*cheADWY, entCES, fepABE, flgGH, fliACGMNP, gmhA.lpcA, gtrAB, iroBCN entA, ratB, shdA, sspH2, shdA, steB sopD*
2011	19CS0430	FLI	Pig	7	*blaTEM-1, aadA1, aadA2, tet.A7 sul3, cmlA1, fosA7*	4	ColRNAI_1, IncI1_1_Alpha, Col440II_1, Col440II_1	126	*cheADWY, entCES, fepABE, flgGH, fliACGMNP, gmhA.lpcA, gtrAB, iroBCN entA, ratB, shdA, sspH2, shdA, steB sopD*
2014	19CS0456	FLI	Pig	1	*fosA7*	0		125	*cheADWY, entCES, fepABE, flgGH, fliACGMNP, gmhA.lpcA, gtrAB, iroBCN entA, ratB, shdA, sspH2, shdA, steB sopD*
2015	19CS0462	FLI	Pig	1	*fosA7*	0		125	*cheADWY, entCES, fepABE, flgGH, fliACGMNP, gmhA.lpcA, gtrAB, iroBCN entA, ratB, shdA, sspH2, shdA, steB sopD*
2008	ERR2195751	PC	Pig	1	*fosA7*	0		95	*sopD2, sseK2*
2008	ERR2195752	PC	Pig	1	*fosA7*	0		95	*sopD2, sseK2*
2008	ERR2195754	PC	Pig	1	*fosA7*	1	Col8282_1	95	*sopD2, sseK2*
2010	ERR2195759	PC	Pig	1	*fosA7*	1	ColRNAI_1	95	*sopD2, sseK2*
2010	ERR2195761	PC	Pig	1	*fosA7*	1	ColRNAI_1	95	*sopD2, sseK2*
2012	ERR2195768	PC	Pig	1	*fosA7*	1	ColRNAI_1	95	*sopD2, sseK2*
2013	ERR2195774	PC	Pig	1	*fosA7*	0		95	*sopD2, sseK2*
2014	ERR2195775	PC	Pig	1	*fosA7*	2	ColRNAI_1, IncX4_1	94	*sopD*
2014	ERR2195777	PC	Pig	1	*fosA7*	1	Col440II_1	94	*sopD2, sseK2*, No seek1 present
2008	ERR2195780	PC	Cattle	5	*aph(6)-Id, aph(3”)-Ib, dfrA14*, sul2, *fosA7*	1	ColRNAI_1	94	*sopD2, sseK2*
2008	ERR2195781	PC	Cattle	1	*fosA7*	1	Col440II_1	95	*sopD2, sseK2*
2009	ERR2195783	PC	Cattle	1	*fosA7*	0		95	*sopD2, sseK2*
2009	ERR2195784	PC	Cattle	1	*fosA7*	0		95	*sopD2, sseK2*
2009	ERR2195785	PC	Cattle	1	*fosA7*	0		95	*sopD2, sseK2*
2010	ERR2195786	PC	Cattle	1	*fosA7*	0		95	*sopD2, sseK2*
2010	ERR2195787	PC	Cattle	1	*fosA7*	0		95	*sopD2, sseK2*
2011	ERR2195788	PC	Cattle	1	*fosA7*	1	ColRNAI_1	95	*sopD2, sseK2*
2014	ERR2195795	PC	Cattle	1	*fosA7*	1	ColRNAI_1	95	*sopD2, sseK2*
2009	ERR2195799	PC	Cattle	1	*fosA7*	0		95	*sopD2, sseK2*
2004	ERR3263622	PC	Pig	1	*fosA7*	1	IncI1_1_Alpha	95	*sopD2, sseK2*
2007	ERR3263629	PC	Human	1	*fosA7*	0		95	*sopD2, sseK2*
2014	ERR3263667	PC	Human	1	*fosA7*	1	ColRNAI_1	95	*sopD2, sseK2*
2014	ERR3263672	PC	Human	1	*fosA7*	1	Col440I_1	95	*sopD2, sseK2*
2018	ERR3264069	PC	Human	1	*fosA7*	1	ColRNAI_1	95	*sopD2, sseK2*
2018	ERR3264070	PC	Human	1	*fosA7*	1	ColRNAI_1	95	*sopD2, sseK2*

*PC: public collection, FLI: our collection.*

**TABLE 3:** ST40 genome information about the antimicrobial resistance genes, plasmid replicons and virulence genes, *n* = 21.

Metadata				Antibiotic resistance genes		Plasmid		Virulence	
			
Year	ID strain	Collection	Sample	N	Gene names	N	Replicon name		N
2010	19CS0413	FLI	Pig	1	*fosA7.3*	5	Col.MGD2._1, Col.Ye4449._1, Col8282_1, ColRNAI_1, IncQ2_1	127	*cheADWY, entCES, fepABE, flgGH, fliACGMNP, gmhA.lpcA, gtrAB, iroBCN entA, ratB, shdA, sspH2, shdA, steB sopD, sseK2*
2010	19CS0427	FLI	Pig	1	*fosA7.3*	7	Col.MGD2._1, Col.Ye4449._1, Col440II_1, Col440I_1, Col8282_1, ColRNAI_1, IncQ2_1	126	*cheADWY, entCES, fepABE, flgGH, fliACGMNP, gmhA.lpcA, gtrAB, iroBCN entA, ratB, shdA, sspH2, shdA, steB sopD, sseK2*
2015	19CS0459	FLI	Environment	2	*qnrB19, fosA7.3*	3	Col8282_1, ColE10_1, IncI1_1_Alpha	127	*cheADWY, entCES, fepABE, flgGH, fliACGMNP, gmhA.lpcA, gtrAB, iroBCN entA, ratB, shdA, sspH2, shdA, steB sopD, sseK2*
2015	19CS0460	FLI	Environment	4	*qnrB19, fosA7.3*	3	Col8282_1, ColE10_1, IncI1_1_Alpha	127	*cheADWY, entCES, fepABE, flgGH, fliACGMNP, gmhA.lpcA, gtrAB, iroBCN entA, ratB, shdA, sspH2, shdA, steB sopD, sseK2*
2008	ERR2195750	PC	Pig	4	*aadA2, tetA, sul1, fosA7.3*	2	Col.Ye4449._1, IncQ2_1	96	*ssp*H2
2008	ERR2195753	PC	Pig	4	*aadA2, tetA, sul1, fosA7.3*	2	Col.Ye4449._1. IncQ2_1	95	*sopD2, sseK2*
2008	ERR2195756	PC	Pig	1	*fosA7.3*	1	ColRNAI_1	95	*sopD2, sseK2*
2010	ERR2195762	PC	Pig	2	*tetA, fosA7.3*	3	Col156_1, IncFIB.A, IncFIC.FII._1	95	*sopD2, sseK2*
2011	ERR2195764	PC	Pig	7	*bla_*TEM–1*,_ aadA2, tetA, dfrA1, sul1, sul2, fosA7.3*	3	Col.Ye4449._1, IncQ2_1, IncI1_1_Alpha	95	*sopD2, sseK2*
2012	ERR2195767	PC	Pig	2	*tetB, fosA7.3*	2	Col.VCM04. _1, ColRNAI_1	79	No *ssa*CDEGHIJ, *ssc*AB, *sse*BCDEFG
2012	ERR2195769	PC	Pig	1	*fosA7.3*	1	ColRNAI_1	95	*sopD2, sseK2*
2013	ERR2195770	PC	Pig	5	*aadA1, tetB, dfrA1, sul1, fosA7.3*	3	IncHI2A_1, IncHI2_1, RepA_1_pKPC	94	No *sse*L
2009	ERR2195782	PC	Pig	8	*bla*_TEM–1,_ *aadA, aph(3”)-Ib, aph(6)-Id, dfrA1, sul1, sul2, fosA7.3*	1	IncI1_1_Alpha	95	*sopD2, sseK2*
2011	ERR2195789	PC	Cattle	1	*fosA7.3*	2	Col440II_1, ColRNAI_1	95	*sopD2, sseK2*
2011	ERR2195791	PC	Wild boar	1	*fosA7.3*	1	IncI1_1_Alpha	95	*sopD2, sseK2*
2013	ERR2195794	PC	Cattle	5	*aadA1, dfrB1, sul1, catB2, fosA7.3*	3	Col440II_1, ColRNAI_1, IncI1_1_Alpha	94	No *sop*D
2007	ERR3263628	PC	Human	1	*fosA7.3*	0		95	*sopD2, sseK2*
2012	ERR3263635	PC	Human	4	*bla*_TEM–1_, *sul3*, *f fosA7.3*, mutation in *gyrA_S83F*	4	Col440I_1, IncFIB.A, IncFIC.FII._1	94	No *ssa*G
2014	ERR3263659	PC	Human	1	*fosA7.3*	1	Col440I_1	95	*sopD2, sseK2*
2014	ERR3263668	PC	Human	4	*aadA2, tetA, sul1, fosA7.3*	3	Col.Ye4449._1, Col440I_1, IncQ2_1	95	*sopD2, sseK2*
2018	ERR3264071	PC	Human	7	*bla*_TEM–1,_ *qnrS1*, *tetM*, *tetA*, *sul2*, *floR*, *fosA7.3*	1	Col440I_1	96	*ssp*H1

*PC: public collection, FLI: our collection.*

**TABLE 4:** ST682 genome information about the antimicrobial resistance genes, plasmid replicons and virulence genes, *n* = 25.

Metadata				Antibiotic resistance genes		Plasmid		Virulence	
			
Year	ID strain	Collection	Sample	N	Gene names	N	Replicon name	N	Genes
2000	19CS0432	FLI	Cattle	0		0		132	*lpfABCDE, sspH1, hsiC1.vipB, cheADWY, entCES, fepABE, flgGH, fliACGMNP, gmhA.lpcA, gtrAB, iroBCN entA, ratB, shdA, sspH2, shdA, steB sopD, sseK2*
2015	19CS0458	FLI	Environment	0		0		132	*lpfABCDE, sspH1, hsiC1.vipB, cheADWY, entCES, fepABE, flgGH, fliACGMNP, gmhA.lpcA, gtrAB, iroBCN entA, ratB, shdA, sspH2, shdA, steB sopD, sseK2*
2008	ERR2195755	PC	Pig	0		1	IncI1_1_Alpha	104	*lpfABCDE, sspH1, entA, ratB, shdA, sspH2, shdA, steB sopD, sseK2*
2011	ERR2195765	PC	Environment	4	*bla*_TEM–1,_ *aadA1, aadA2, sul3, cmlA1*	2	IncX1_1, IncX3_1	104	*lpfABCDE, sspH1, entA, ratB, shdA, sspH2, shdA, steB sopD, sseK2*
2012	ERR2195766	PC	Pig	0		0		104	*lpfABCDE, sspH1, entA, ratB, shdA, sspH2, shdA, steB sopD, sseK2*
2013	ERR2195772	PC	Pig	0		1	Col156_1	104	*lpfABCDE, sspH1, entA, ratB, shdA, sspH2, shdA, steB sopD, sseK2*
2013	ERR2195773	PC	Pig	0		0		104	*lpfABCDE, sspH1, entA, ratB, shdA, sspH2, shdA, steB sopD, sseK2*
2014	ERR2195778	PC	Pig	0		0		104	*lpfABCDE, sspH1, entA, ratB, shdA, sspH2, shdA, steB sopD, sseK2*
2014	ERR2195779	PC	Pig	0		0		104	*lpfABCDE, sspH1, entA, ratB, shdA, sspH2, shdA, steB sopD, sseK2*
2014	ERR2195796	PC	Cattle	0		0		104	*lpfABCDE, sspH1, entA, ratB, shdA, sspH2, shdA, steB sopD, sseK2*
2008	ERR3263630	PC	Human	0		0		104	*lpfABCDE, sspH1, entA, ratB, shdA, sspH2, shdA, steB sopD, sseK2*
2012	ERR3263634	PC	Human	0		0		104	*lpfABCDE, sspH1, entA, ratB, shdA, sspH2, shdA, steB sopD, sseK2*
2012	ERR3263636	PC	Human	0		0		104	*lpfABCDE, sspH1, entA, ratB, shdA, sspH2, shdA, steB sopD, sseK2*
2012	ERR3263637	PC	Human	0		0		104	*lpfABCDE, sspH1, entA, ratB, shdA, sspH2, shdA, steB sopD, sseK2*
2012	ERR3263638	PC	Human	0		2	Col8282_1, ColRNAI_1	104	*lpfABCDE, sspH1, entA, ratB, shdA, sspH2, shdA, steB sopD, sseK2*
2012	ERR3263639	PC	Pig	0		0		104	*lpfABCDE, sspH1, entA, ratB, shdA, sspH2, shdA, steB sopD, sseK2*
2013	ERR3263643	PC	Human	1	*sul2*	1	ColRNAI_1	104	*lpfABCDE, sspH1, entA, ratB, shdA, sspH2, shdA, steB sopD, sseK2*
2013	ERR3263651	PC	Human	0		0		104	*lpfABCDE, sspH1, entA, ratB, shdA, sspH2, shdA, steB sopD, sseK2*
2013	ERR3263652	PC	Poultry	0		1	IncI1_1_Alpha	104	*lpfABCDE, sspH1, entA, ratB, shdA, sspH2, shdA, steB sopD, sseK2*
2013	ERR3263655	PC	Human	0		0		104	*lpfABCDE, sspH1, entA, ratB, shdA, sspH2, shdA, steB sopD, sseK2*
2014	ERR3263656	PC	Human	0		0		104	*lpfABCDE, sspH1, entA, ratB, shdA, sspH2, shdA, steB sopD, sseK2*
2014	ERR3263669	PC	Human	0		0		104	*lpfABCDE, sspH1, entA, ratB, shdA, sspH2, shdA, steB sopD, sseK2*
2014	ERR3263671	PC	Human	0		0		104	*lpfABCDE, sspH1, entA, ratB, shdA, sspH2, shdA, steB sopD, sseK2*
2014	ERR3263676	PC	Human	7	*bla*_TEM–1,_ *aadA1, aph(3”)-Ib, aph(6)-Id, dfrA1, sul1, sul2*	1	IncI1_1_Alpha	104	*lpfABCDE, sspH1, entA, ratB, shdA, sspH2, shdA, steB sopD, sseK2*
2014	ERR3263682	PC	Human	0		1	IncFII.pCRY._1_pCRY	104	*lpfABCDE, sspH1, entA, ratB, shdA, sspH2, shdA, steB sopD, sseK2*

*PC: public collection, FLI: our collection.*

In the public collection, ST39 sequenced carried the *fosA7* gene (Table 2). Only the sequence ERR2195780 (cattle) carried another ARGs (*aph(6)-Id, aph(3”)-Ib, dfrA14*, and *sul2*). No quinolone resistance genes were identified in the ST39. All ST40 sequences presented *fosA7.3*, and 64.7% (11/17) of them carried other ARGs conferring resistance to β-lactam-antibiotics, aminoglycosides, tetracycline, trimethoprim, sulfonamide, quinolone, and/or phenicol (Table 3). ST40 strain ERR2195782 (pig) carried the highest number of ARGs (*n* = 7) *bla*_TEM–1_, *aadA1, aph(6)-Id, aph(3”)-Ib, dfrA1, sul1* and *sul2* (Table 3). Twelve percent (3/23) of the ST682 isolates carried at least one ARG (Table 4). These genes encoded resistance to β-lactam-antibiotics, aminoglycoside, trimethoprim, and sulfonamide. The most resistance isolate in this ST was ERR3263676 (pig) with *bla*_TEM–1,_ *aadA1, aph(6)-Id, aph(3”)-Ib, dfrA1, sul1* and *sul2* genes.

### Plasmids and the Relation With ARGs

Twenty-one different plasmid replicons (Supplementary Table 3) were found in the 80 strains. Sixty percent (48/80) of these strains were positive to at least one plasmid replicon. ColRNAI_1 was the most frequently detected replicon (30% of the strains (24/80)) followed by IncI1_1_Alpha (13.4% (11/80)).

In the FLI collection, plasmid replicons were found in ST39 and ST40, but not in ST682. ST40 carried a high number and diversity of plasmids replicons (Table 3). ColRNAI_1 was most frequently found, especially in ST39 (Table 2).

In the public collection, plasmid replicons were detected among the strains independently of their ST. 60% of ST39 sequences carried at least one and ColRNAI was the most frequently found (Table 2). Almost all ST40 isolates (94.11%) carried at least one plasmid replicon (Table 3), and up to four different replicons were found in the strains. In ST682 isolates, 34.8% of the sequences carried a plasmid replicon; IncI1_1_Alpha was most frequently detected (Table 4).

#### IncI1 Plasmid

**FIGURE 1:**
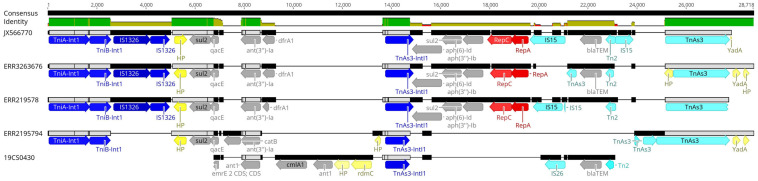
Multiple alignments of plasmid sequences belonging to the IncI1 ST26 CC-2 in the 28,781 bp region. Dark blue: Integron genes, yellow: Hypothetical protein gene (HP) and other genes, gray: Antimicrobial resistance genes, red: Plasmid replicase gen, light blue: other mobile elements like transposons (Tn) and insertion islands (IS).

Eleven sequences with IncI1_1_Alpha replicon were classified in different plasmid sequence types (3, 26, 80, 134, and unknown). Four of them belonged to the same plasmid ST26 and identical clonal complex 2. They were carried by four strains (19CS0430, ERR2195782, ERR2195794, and ERR3263676), which belonged to different sources and STs (Table 5). These four plasmids were compared with a published plasmid sequence of the same ST and clonal complex collected from a German pig in 2010 (JX566770). Multiple plasmid alignments indicated high percentage identity between them, except for a segment located in a region of around 28,781 bp. This region carried different ARGs (*bla*_TEM–1,_ *aph(3”)-Ib, aph(6)-Id, ant(3”)-Ia, dfrA1, sul1 sul2*, and *qacEdelta1)* surrounded by mobile genetic elements (MGEs) (Figure 1). This region could be separated into two parts: The first part from 0 to 15,000 bp was assigned to class 1 Integron (Tn1) with ARGs (*sul2, qacE, ant(3”)-Ia, dfrA1*) (Figure 1). It was identical to JX566770 (pig), ERR3263676 (human) and ERR2195782 (pig) (Figure 1 and Table 5). The other two sequences ERR2195794 (cattle) and 19CS0430 (pig) carried some parts of the Tn1 and other ARGs (Figure 1 and Table 5). Moreover, the second part of the sequence contained ARGs, plasmid replicase genes, and other MGEs (Figure 1). Only the first three sequences (JX566770, ERR3263676, and ERR2195782) were similar in this second part.

**TABLE 5:** Percentage of identity between sequences with IncI1 ST26 CC-2 plasmid.

Isolate	ST	Sample	Collection	Year	JX566770	ERR3263676	ERR219578	ERR2195794	19CS0430	Resistance genes region 28,000bp
JX566770	39	Pig	PC	2010	0	93.0%	95.6%	39.8%	30.8%	*sul2, qacEdelta1, dfrA ant(3”)-Ia, aph(3”)-Ib, aph(6)-Id, blaTEM*
ERR3263676	682	Human	PC	2014	93.0%	0	94.3%	44.7%	28.0%	*sul2, qacEdelta1, dfrA, ant(3”)-Ia, aph(3”)-Ib, aph(6)-Id, blaTEM*
ERR2195782	40	Pig	PC	2009	95.6%	94.3%	0	40.2%	30.3%	*sul2, qacEdelta1, dfrA, ant(3”)-Ia, aph(3”)-Ib, aph(6)-Id*
ERR2195794	40	Cattle	PC	2013	39.8%	44.7%	40.2%	0	23.3%	*sul2, qacEdelta1, catB, ant(3”)-Ia*
19CS0430	39	Pig	FLI	2011	30.8%	28.0%	30.3%	23.3%	0	*ant(3”)-Ia cmlA1, blaTEM*

*These percentages correspond to a region of 28,781 bp located in all plasmids. PC: public collection, FLI: our collection.*

### SPI and Virulence-Associated Genes

SPI-1 to 6, SPI-9, SPI-11, SPI-12, CP54, and C63PI were detected in all strains. SPI-8 was only present in ST682 strains of both collections (Supplementary Table 4). Up to 132 virulence factors were found, but the number varied between STs and collections (Supplementary Table 5).

In the FLI collection, each strain showed between 125 and 132 virulence-associated genes. The genes *gtrA* and *gtrB* were identified in all strains of our collection. They belong to SPI-16 and are involved in serotype conversion through glycosylation of O-antigen. ST682 carried more virulence-associated genes than the other STs; *lpfABCDE*, *sspH1* (encoded to proteins for a long polar fimbria), and *hsiC1.vipB* (Type VI secretion protein) (Table 4) were detected. *sopD2* (endosome/lysosome) and *sseK2* (biofilm formation) were present in all the sequences belonging to ST39 and ST40 (Table 2 and Table 3). Besides, *flhC*In (Flagellar transcriptional regulator FlhC) was found exclusively in ST40 (Table 3).

In the public collection, between 79 and 104 virulence genes were identified (Table 4). ST682 carried more virulence-associated genes than other STs. This ST carried *lpfABCDE*, *sspH1* (encoded to proteins for a long polar fimbria), *entA* (iron-regulated promoter), *ratB* (CS54 island), *shdA* (biofilm formation), *sspH2* (Type III effector), *shdA* (fibronectin) *and steB* (biofilm formation) (Table 4). Genes *sopD2* (endosome/lysosome) and *sseK2* (biofilm formation) were present in all ST39 and ST40 sequences, but not in ST682 (Table 4).

### Phylogenetic Analysis and Comparison of Database (All Strains)

**FIGURE 2:**
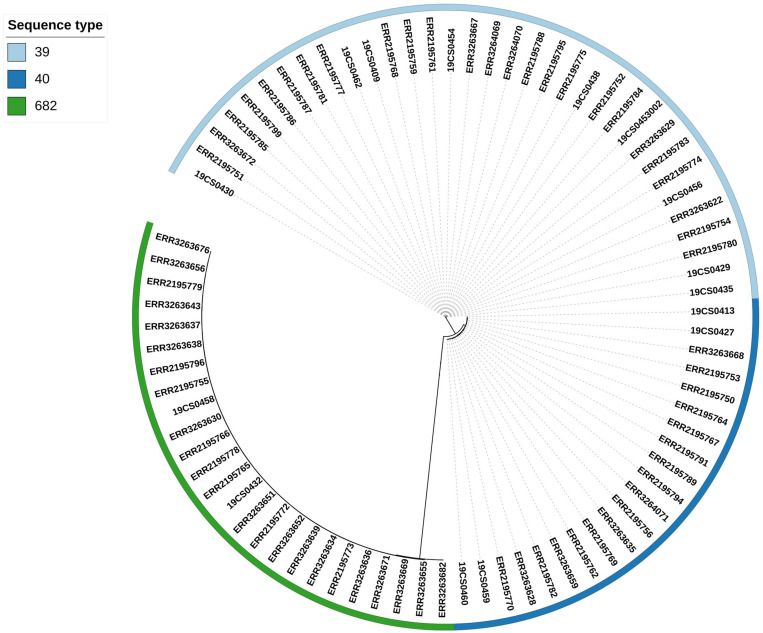
Phylogenetic SNP tree of the 80 S. Derby strains. The colored zone alongside the tree represents the ST of the isolates: ST39 (bright blue), ST40 (dark blue), and ST682 (green).

Three clusters were obtained by single nucleotide polymorphisms (SNPs) analysis (Supplementary Table 6) of the 80 isolates (15 *S*. Derby from our collection and 65 *S*. Derby sequences from the public collection) (Figure 2). The clusters were consistent with the ST profiles ST39, ST40, and ST682 (Figure 2). The difference in SNPs average between STs depends on their genetic distance. ST39 and ST40 are closer with an average of 2,578 SNPs, and both were distant from the strains belonging to ST682 with an average of 18,416 SNPs and 17,660 SNPs, respectively.

### ST39

**FIGURE 3:**
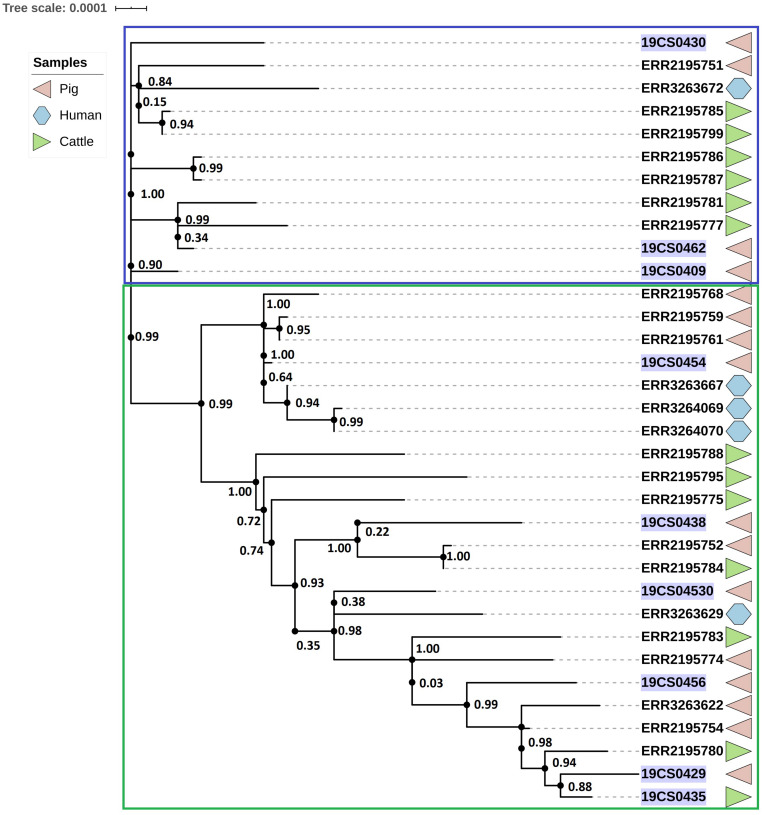
Phylogenetic SNP-based tree of S. Derby ST39 strains. Clusters are labeled in colored rectangles: cluster 1 (blue) and cluster 2 (green). The colored figures alongside the tree represent the source of the isolates: pig (pink), human (blue), and cattle (green). FLI isolates marked in purple.

The ST39 cluster is composed of 34 strains (FLI *n* = 9; PC *n* = 23) (Figure 3). The average of SNPs between the ST39 strains was 47 with a standard deviation (SD) of 18 SNPs. These samples were taken from pigs (*n* = 18), cattle (*n* = 11), and humans (*n* = 5) over a period from 2005 to 2015. It is divided into two clades. Samples of both collections (purple marked: FLI collection, Figure 3) and source (pigs, human and cattle samples) share the two clades. The first clade (blue square, Figure 3) carried 11 isolates; they are separated by an average of 23 SNPs with an SD of 9 SNPs. The second clade (green square, Figure 3) carried 23 isolates with an average distance of 47 SNPs with an SD of 9 SNPs.

### ST40

**FIGURE 4:**
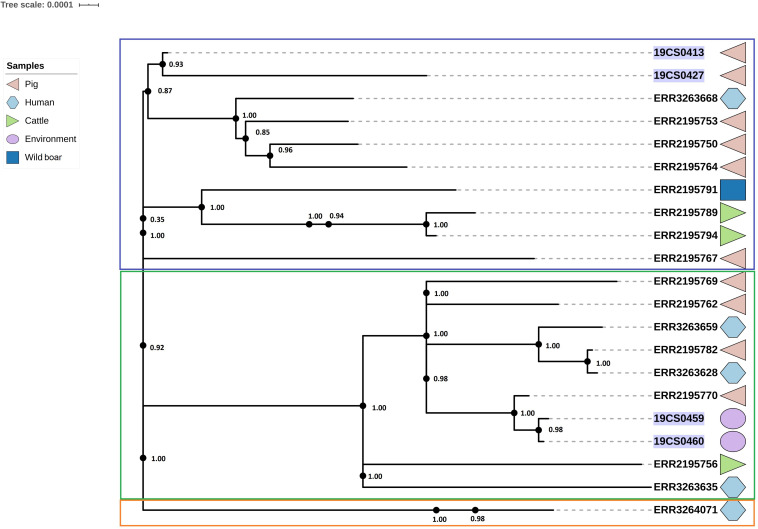
Phylogenetic SNP tree of S. Derby ST40 strains. Clusters are labeled in colored rectangles: cluster 1 (blue), cluster 2 (green) and cluster 3 (orange). The colored zone alongside the tree represents the source of the isolates: pig (pink), human (blue) and cattle (green), environment (purple), and wild type (dark blue). FLI isolates marked in purple.

The ST40 cluster contained 21 strains (FLI *n* = 4; PC *n* = 17) (Figure 4). The average SNPs distance between these strains was 118 with an SD of 42 SNPs. The samples were obtained from pigs (*n* = 9), cattle (*n* = 2), humans (*n* = 5), wild boar (*n* = 1), and the environment of pig slaughterhouse (*n* = 2). Three clusters were found (Figure 4); the first (blue square, Figure 4), and the second (green square, Figure 4) carried 10 isolates, respectively, and they belonged to both collections. The third carried only one human isolate of the public collection. The first clade had isolates of pig, human, cattle, and wild boar with an average of 94 SNPs with an SD of 34 SNPs. In the second cluster (green square, Figure 4) were grouped samples of humans, cattle, pigs, and environmental samples of pig slaughterhouses. The distance between the isolates of this cluster had an average of 70 SNPs with a SD of 31 SNPs.

### ST682

**FIGURE 5:**
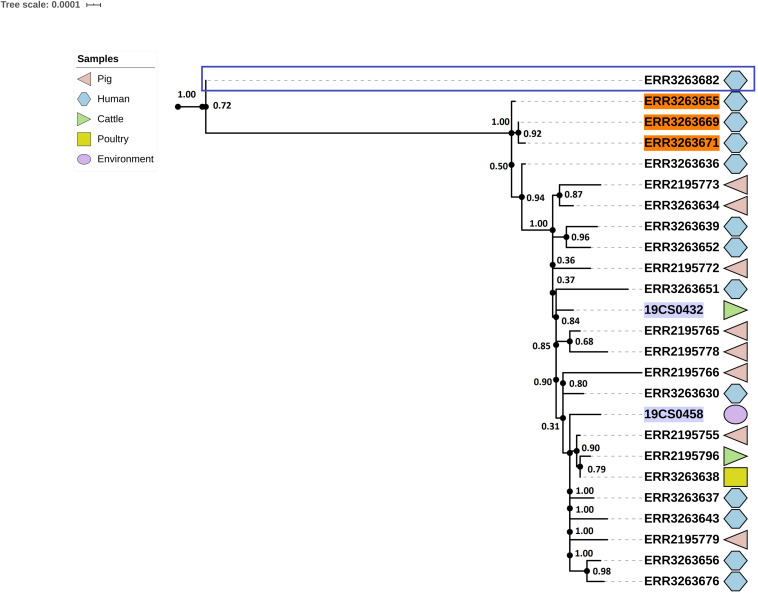
Phylogenetic SNP-based tree of S. Derby ST682 strains. Cluster 1 (blue rectangle) and cluster 2 (rest of the sequences, not labeled). The colored zone alongside the tree indicates the source of the isolates: pig (pink), human (blue) cattle (green), environment (purple), and poultry (yellow). FLI isolates marked in purple. Human isolates of the 2013 outbreak are highlighted in orange.

Twenty-five strains (FLI *n* = 2; PC *n* = 23) were assigned as ST682; this ST presents a homogeneous tree (Figure 5). The average of SNPs distance between these strains was 29 with a SD of 25 SNPs. Two clusters were defined; however, one of them contained only one human isolate of the public collection (blue square, Figure 5).

The average of SNPs in the second clade was 22 with SD of 7 SNPs. The samples were taken from pigs, the environment of a pig slaughterhouse, cattle, poultry, wild boar and humans (*n* = 13) from both collections. Three human samples (lighted in orange, Figure 5) of the *S.* Derby outbreak in 2013 are also in this clade.

## Discussion

Although *S.* Derby is an important serovar, its genetic diversity has not been extensively investigated, which results in a limited public collection of sequence data of *Salmonella* Derby from pig and cattle in Germany. Consequently, this publication does not claim to be representative for the whole country. However, with this study, we increased the public sequence data and with the comparison of both collections, we intended to get more information about the diversity of *S.* Derby in Germany using comprehensive genomic analyses.

Three STs (ST39, ST40, and ST682) were analyzed. The predominant ST was ST39, followed by ST40 and ST682. They were commonly found in other countries like France (Sévellec et al., 2018) and China (Zheng et al., 2017) and were associated with pigs (Hauser et al., 2011; Zheng et al., 2017; Sévellec et al., 2018) and humans (Sévellec et al., 2020). For example, ST39 is predominantly associated with pork (Sévellec et al., 2019), and ST682 was found in humans and some chicken samples as part of a *S.* Derby outbreak taking place in Germany between 2013 and 2014 (Simon et al., 2018). In France, ST682 and ST40 were responsible for most human cases (Sévellec et al., 2020). In our study, as in the literature, most of our sequences belonged to pig or environmental pig-related samples (52%) and humans (29%). We observe same STs as previously reported in Germany and Europe. Interestingly, we did not find cattle-related S. Derby ST information in the literature. This could be because of the low prevalence of *Salmonella* in cattle in Germany (1%) (BVL, 2019) and in Europe (3.86%) (EFSA, 2018) compared with poultry and pigs. However, *Salmonella* in cattle has a significant zoonotic risk (Hoelzer et al., 2011) of causing infections in humans (Hsu et al., 2019; Kudirkiene et al., 2020). Besides, it causes economic losses because of mortality, and costs associated with infection control and treatment (Hoelzer et al., 2011; Hsu et al., 2019). In this study, cattle samples comprise 18.7% of the total sequence data, whereby three STs were found and most strains belonged to ST39.

In a recent study in France, 61% of the *S.* Derby isolates carried antimicrobial resistance genes (ARGs), but 41% of these samples belonged to ST71, a pansusceptible ST (Sévellec et al., 2018). In Germany, 25% of the pig and human *S*. Derby isolates carried ARGs (Hauser et al., 2011). In our study at least one ARGs was found in 72.5% (58/80) of both sequence collections. This high prevalence is probably due to the frequent detection of fosfomycin ARGs (*fosA7, fosA7.3)*, that was found in all ST39 and ST40 sequences, the same result as in French isolates (Sévellec et al., 2020). Note that these fosfomycin genes were only recently included in the ResFinder database and, possibly therefore, had not been reported prior to 2020 (Sévellec et al., 2020).

Other important ARGs found in French isolates were *aadA2, sul1*, and *tetA* (Sévellec et al., 2018) which was also found in Germany (*tetB*, *sul1*, *aadA2*, and *int)* (Hauser et al., 2011). Moreover, in the United States, *sul1* (32.5%), *tetR* (28.5%), and *tetA* (24%) were the most common ARGs detected in *Salmonella* spp. These results agree with the results of this study, where tetracycline (*tetB, tetM, tetG, tetA*; 11.2%), sulfonamide (*sul1, sul2, sul3;* 17.5%) and aminoglycosides (*aadA1, aadA2, aph(6)-Id, aph(3”)-Ib;* 13.7%) were the most prevalent. Other ARGs associated with resistance to β-lactam-antibiotics (*bla*_TEM–1_; 8.7%), trimethoprim (*dfrA14, dfrA1, dfrB1*; 7.5%), phenicol (*cmlA1, floR, catB2*; 5%) and fluoroquinolone (*qnrS1, qnrB*; 3.7%) were also found. Additionally, 18.7% of the isolates carried ARGs from other antibiotic families. This prevalence is slightly higher than that reported for *S*. Derby isolates in pigs (11.8%), pig carcasses (15.3%) and calf carcasses (14.3%) in the EU (EFSA, 2019).

The ARGs distribution between the STs in both collections was similar. ST40 presented the most resistant isolates; 57.1% of these ST isolates carried ARGs against six different antimicrobial families (β-lactam-antibiotics, aminoglycosides, tetracycline, trimethoprim, sulfonamide, quinolone, and/or phenicol). Only two ST39 strains carried other genes than those coding for resistance against fosfomycin. Moreover, only three ST682 strains carried ARGs (β-lactam-antibiotics, aminoglycoside, trimethoprim, and sulfonamide). These results are analogous to the results in France, where ST40 carried 86% of the detected ARGs (Sévellec et al., 2018). However, in our study, antibiotic resistance was found with a lower prevalence in ST39 and ST682, as opposed to the study in France, where no ARGs were found in these STs (Sévellec et al., 2018). In conclusion, the overall prevalence and diversity of ARGs of antibiotic families identified in our study are in good agreement with previous studies in other countries.

Sixty percent (48/80) of ST40 strains were positive to at least one plasmid replicon. In both collections, ST40 carried a high number of diverse plasmid replicons. ST682 carried the smallest number of replicons in the public collection, and none were detected in the strains of our collection. Of the 21 different replicons found, ColRNAI_1 was the most frequently detected in both collections, where 62.5% corresponded to ST39 isolates. In the United States, this replicon was detected in 43% of the *Salmonella* spp. isolates (Tyson et al., 2017). ColE10_1, another replicon of the Col Family, which is usually detected in *Salmonella* spp., is highly associated with the spread of *qnrS1* and *qnr*B19 genes (Partridge et al., 2018; Rozwandowicz et al., 2018). This is in agreement with the results of our study, where the only two isolates that carried *qnrB19* (Table 3) were those related to the ColE10_1 replicon. IncI1_1_Alpha was the second most frequently found replicon; it was detected in 13.4% (11/80) of the isolates of different STs. This replicon is broadly distributed in *Salmonella* spp. and other clinically important enteric bacteria. This plasmid is highly persistent, owing to the minimal metabolic costs on the host strains (Kaldhone et al., 2019). Besides, it has a great potential to transmit and disseminate ARGs against aminoglycosides, tetracycline, quinolones (Rozwandowicz et al., 2018) and β-lactamases (Partridge et al., 2018) among enteric pathogens (Kaldhone et al., 2019). In this study, all IncI1 positive strains carried multiple ARGs. To determine its possible dissemination among the isolates, the replicons were subtyped by pMLST Web tool. Four of the replicons belonged to ST26 clonal complex 2. This agrees with the previous observations that this ST is the most commonly found in IncI plasmids (Kaldhone et al., 2019). The comparison between our samples and the plasmid of *S.* Derby JX566770 of the identical ST and complex clone (Bleicher et al., 2013) shows that all plasmids were almost identical except for one region where ARGs were surrounded by mobile genetic elements. Two of the isolates (pig and human origin) were almost identical to plasmid JX566770, whereas for two others (pig and cattle) the number and type of ARGs were different (Figure 1 and Table 5). It is important to note that this plasmid has also been found in samples of both collections with distribution among different sources (pig, human, and cattle) in different years and different STs. This is also remarkable because the ST39 and ST682 strains analyzed in our study did not carry many ARGs and the few strains that did also carried IncI replicon. We thus see that the resistance in the serovar is not only a function of ST (ST40 carried most of the resistance genes) but that multidrug-resistance may also be carried by plasmids like IncI1.

Pathogenicity genes are clustered in the *Salmonella* pathogenicity islands (SPIs) (Marcus et al., 2000). In total, 23 SPIs have been described and characterized (Espinoza et al., 2017). SPI-1 to SPI-5 are common to all serovars of *S. enterica*, while the rest are distributed among different serovars (Espinoza et al., 2017). In this study, all the strains carried SPI-1 to SPI-5 which was also found in France (Sévellec et al., 2018). SPI-8 was detected in all ST682 strains; it is widely distributed among *Salmonella* serovars and improves bacterial fitness of typhoid serovars in the human gut (Espinoza et al., 2017). In the case of other virulence-associated genes, the samples of our collection carried more of these genes than the sequence data of the public collection. This was the only notable difference between the isolates of both collections. The virulence-associated genes detected are important in the bacterial chemotaxis (*cheADWY)*, for surface proteins as O antigen (*gtrAB*) and flagellum proteins (*flgGH, fliACGMNP).* In the strains of our collection, genes encoding important proteins for the metabolism and the uptake of iron have been found (i.e.: *fepABE* (iron-regulated) and *iroBCN* (salmochelin siderophore system)). They play an important role in the competition of *Salmonella* spp. for nutrients and colonization in the intestinal epithelial cells (Kaldhone et al., 2019).

Three clusters consistent with the ST profiles ST40, ST39, and ST682 were constructed based on single nucleotide polymorphisms (SNPs). ST40 and ST39 were closely related (3,216 SNPs) and distant to the ST682 strains (21,803 SNPs). This result is similar to the situation in a French study where ST40 and ST39 genetically related with an average of 3,962 SNPs and ST682 was genetically distant with an average of 33,961 SNPs (Sévellec et al., 2018). In Germany, the difference in the average distance between ST682 and both ST40 and ST30 was 40,000 SNPs (Simon et al., 2018). Within the genomes from the same ST isolates, a difference of fewer than 300 SNPs was found in a previous study (Sévellec et al., 2018). In our study, we have found a lower difference (less than 133 SNPs) between the genomes of the same STs. Importantly, all species from both collections share clades, showing close relation among them.

The comparison of our collection with the public collection data adds information that will lead to a better understanding of the diversity of *Salmonella* Derby in Germany, and highlights some key differences and similarities: ST39, ST40, and ST682 are present in *S.* Derby in Germany; they are distributed in pigs, cattle, and humans, where no difference between the species was found. Resistance determinants were found in the three STs, but especially in ST40, where most of the ARGs and replicons were found. In the case of plasmids, the distribution of IncI shows that this plasmid circulated in this serovar carrying ARGs. The resistance not only depends on the ST but also on the plasmid, which can transfer the resistance among isolates of different species. *S.* Derby was most studied in pigs, but special attention must be paid to cattle. This animal is an important factor of *Salmonella* infections in humans, and no information about ST in *S.* Derby has been found. In this study, cattle samples were closely related to pigs and humans, showing possible contact between species, which could also play a role in the spread of *S.* Derby. In conclusion, this study contributes to a growing body of knowledge about *S.* Derby and its distribution in human and animal reservoirs in Germany and their multidrug-resistant plasmids. However, it is of the utmost importance that the public database of *S.* Derby sequences be increased by further studies, because this kind of information will help to detect and ultimately limit the spread of *S.* Derby, and in particular its multidrug-resistant variants.

## Data Availability Statement

The original contributions presented in the study are publicly available. Sequencing data of this study can be found at https://www.ncbi.nlm.nih.gov/bioproject/646595. The names of the repository/repositories and accession number(s) can be found in the article/Supplementary Material.

## Author Contributions

BGS and RF: contributed to conception and design of the study. BGS: performed the laboratory work and wrote the manuscript. BGS and SGS: bioinformatic analysis. DM and RF: provided the samples. HT and HO: review and help in writing the manuscript. All authors contributed to manuscript revision, read, and approved the submitted version.

## Conflict of Interest

The authors declare that the research was conducted in the absence of any commercial or financial relationships that could be construed as a potential conflict of interest.

## Footnotes

1 https://enterobase.warwick.ac.uk/species/index/senterica

2 https://github.com/raymondkiu/fastq-info/blob/master/fastq_info_3.sh

3 https://pubmlst.org/plasmid/

4 https://www.ncbi.nlm.nih.gov/bioproject/PRJEB23440/

5 https://www.ebi.ac.uk/ena/browser/view/PRJEB30317

## Supplementary Material

Supplementary Table 1 Genome accession and metadata for public sequence collection.

Supplementary Table 2 Resistance genes detected in both collections.

Supplementary Table 3 Plasmid replicons detected in both collections.

Supplementary Table 4 Salmonella pathogenic island (SPI) detected in both collections.

Supplementary Table 5 Virulence genes detected in both collections.

Supplementary Table 6 Single nucleotide polymorphisms (SNPs) analysis.

Supplementary Table 7 Genome accession and metadata for our sequence collection.
